# Preparative Separation of Procyanidins from Cocoa Polyphenolic Extract: Comparative Study of Different Fractionation Techniques

**DOI:** 10.3390/molecules25122842

**Published:** 2020-06-19

**Authors:** Said Toro-Uribe, Miguel Herrero, Eric A. Decker, Luis Javier López-Giraldo, Elena Ibáñez

**Affiliations:** 1 School of Chemical Engineering, Food Science & Technology Research Center (CICTA), Universidad Industrial de Santander, Carrera 27, Calle 9, Bucaramanga 68002, Colombia; saidtorouribe@gmail.com (S.T.-U.); ljlopez@uis.edu.co (L.J.L.-G.); 2 Foodomics Laboratory, Institute of Food Science Research (CIAL, CSIC-UAM), Nicolás Cabrera 9, 28049 Madrid, Spain; m.herrero@csic.es; 3 Chenoweth Laboratory, Department of Food Science, University of Massachusetts, 100 Holdsworth Way, Amherst, MA 01003, USA; edecker@foodsci.umass.edu

**Keywords:** cocoa polyphenolic extract, procyanidins, fractionation, preparative separation, antioxidant activity

## Abstract

To provide further insight into the antioxidant potential of procyanidins (PCs) from cocoa beans, PC extract was fractionated by several methodologies, including solid phase extraction, Sephadex LH-20 gel permeation, and preparative HPLC using C18 and diol stationary phases. All the isolated fractions were analyzed by UHPLC-QTOF-MS to determine their relative composition. According to our results, classical techniques allowed good separation of alkaloids, catechins, dimers, and trimers, but were inefficient for oligomeric PCs. Preparative C18-HPLC method allowed the attainment of high relative composition of fractions enriched with alkaloids, catechins, and PCs with degree of polymerization (DP) < 4. However, the best results were obtained by preparative diol-HPLC, providing a separation according to the increasing DP. According to the mass spectrometry fragmentation pattern, the nine isolated fractions (Fractions II–X) consisted of exclusively individual PCs and their corresponding isomers (same DP). In summary, an efficient, robust, and fast method using a preparative diol column for the isolation of PCs is proposed. Regarding DPPH^•^ and ABTS^•+^ scavenging activity, it increases according to the DP; therefore, the highest activity was for cocoa extract > PCs > monomers. Thereby, cocoa procyanidins might be of interest to be used as alternative antioxidants.

## 1. Introduction

Procyanidins (PCs) are one of the subclasses of flavonoids which consist of flavan-3-ols units such as (+)-Catechin and/or (–)-Epicatechin [1] linked through C_4_ → C_8_ bonds but also extend through C_4_ → C_6_ linkages; both are referred to as B-type PCs [2,3,4]. Additionally, the flavan-3-ol units can also be doubly linked by a C_4_–C_8_ bond and an additional ether bond formed between C_2_ and O_7_ (A-type procyanidins) [5]. These flavan-3-ols compounds can also be esterified with gallic acid and glucose moieties [6,7], therefore increasing the structural diversity of PCs.

These compounds are relevant to human diet since they are present in different foodstuffs, such as tea, grape, berries, apple, fruit juices, and cocoa-based products [1,8,9,10,11], among others. In general, the molecular weight of PCs is expressed as degree of polymerization (DP). According to their DP, PCs are classified in monomers (DP = 1), oligomers (DP = 2–10), and polymers (DP > 10) [9]. Previous works have reported a DP up to 12, 17, and 20 for cocoa, cider apple skin, and litchi pericarp, respectively [12,13,14]. The importance of these compounds is related to their functional bioactivities [15]. For instance, PCs have a protective and nutritional role in plants [16], influence the development of flavors and colors of the fruits or food product [17,18], and show in vivo and in vitro bioactivities and absorption [19,20], acting as protective agents for cancer and increasing the antioxidant status of human organism, which was widely discussed by Dillard and German [21].

However, studies focusing on functional activities, dietary effects, interaction with proteins, changes occurring under gastrointestinal conditions, metabolic fate, and cytotoxic actions of individual oligomeric procyanidins are poorly developed. In fact, numerous works include the use of polyphenolic extracts [22,23,24,25], catechins [26], and procyanidins such as dimers [27], trimers [28], tetramers [29,30], and pentamers [31], but studies that include higher molecular weight procyanidins (e.g., hexamers to decamers and polymers) is limited mainly due to high production costs and lack of commercial standards. 

Various classical approaches have been used to fractionate procyanidins. For example, catechins and alkaloids have been fractionated by liquid–liquid separation using a solvent system consisting of different polarity solvents (e.g., methanol–ethyl acetate mixtures), moderately polar (e.g., methylene chloride), non-polar solvents (e.g., chloroform), and polar protic solvents (e.g., methanol, *n*-butanol, and water) [32,33,34]. Size exclusion chromatography using Toyopearl HW-40/50 or Sephadex LH-20 eluting with aqueous acetone, aqueous alcohol, urea, and their combinations have been commonly used [4,35,36]; for instance, Yanagida et al. [37] used Toyopearl HW-40F in the presence of aqueous acetone and 8 M urea, achieving good separation of catechin monomers, procyanidin B2, and procyanidin C1, but the remaining fractions consisted of enriched oligomeric compounds. Similar results using polyethylene glycol resin column were achieved by Sasaki et al. [38], who obtained fractions of mixed procyanidins. Chu et al. (2018) [39] compared the separation efficacy of procyanidins and flavonoid glycosides using six different macroporous resins. Other classical techniques such as thin-layer chromatography and column chromatography have been reported but several disadvantages have been identified, such as time-consuming, secondary pollution, complex process, irreversible adsorption on the solid-phase materials, low yield, and high-cost [40].

Recently, centrifugal partitioning chromatography (CPC), based on the partitioning of the solutes through the mixing of two immiscible phases, has been suggested since it allows higher sample input, high recoveries, and high repeatability [41]. Overall, there are several reports using CPC and its related techniques (e.g., counter-current chromatography, high-speed countercurrent chromatography, and flash counter current chromatography) for separation of alkaloids, catechins, dimers, and enriched oligomeric procyanidin fractions (e.g., trimers and tetramers) [42,43]. The main drawbacks include an incomplete separation of flavonoids because of their similar partition coefficient (K) and the need for several separations in series, or combination with preparative HPLC, to achieve a high degree of purification. In addition, longer run time, up to 9 h [44], and no complete elution of flavonoids with K greater than 2, which can remain on the coil of the apparatus, are observed [45]. Consequently, fractionation of procyanidins from plant extracts is still a challenge. Furthermore, the selection of a method is a critical factor since each plant source has its particular chemical profile, and its stereochemical diversity affects the degree of separation achieved [42].

Normal-phase HPLC separation of procyanidins occurs according to the increase of molecular weight and has been previously assayed with dichloromethane–methanol–formic acid–water as eluent [46]. In this case, the separation of procyanidins is limited to medium DP values; moreover, the use of chlorinated solvents is considered hazardous and considerably limits the fractionation scale-up [47]. Better resolution and separation were obtained by Kelm et al. [48] for cocoa procyanidins; in fact, they could separate alkaloids and procyanidins from monomer to tetradecamers in analytical scale, and up to heptamers in semipreparative scale. The latter was conducted with flow rate at 55 mL/min for 70 min. However, large-scale fractionation of high oligomeric procyanidins (DP > 7) using reverse-, normal- or HILIC-phase, together with structural characterization of the isolated fractions and reduction of run time analysis and cost of the isolation process, deserves further research. 

Thereby, this work aims not only to compare different fractionation procedures for procyanidins (with different degree of polymerization) from cocoa polyphenolic extract but also to propose a robust and easy procedure for allowing high degree of purification in complex natural samples. It is expected that either high polar analytes or small molecular compounds interact with the functional groups of stationary phases, thus establishing multimodal retention mechanism such as hydrogen-bonding, hydrophobic, and hydrophilic interactions. Furthermore, specific retention times for procyanidins can be enhanced. For this purpose, chemical characterization using UHPLC-QTOF-MS and antioxidant activity by both DPPH^•^ and ABTS^•+^ assays were carried out. In addition, various separation conditions such as temperature, solid–liquid ratio, gradient, and run time were optimized for both semi-preparative C18-reverse phase and diol columns. Moreover, the relative composition obtained was compared with classical separation methodologies involving the use of solid-phase separation and gel permeation chromatography on a Sephadex LH-20 column. The correct fraction of PCs can lead to obtaining individual procyanidins for further studies focusing on functional activities, interaction, and changes of procyanidins in different in vivo and in vitro systems.

## 2. Results

In this study, a green procedure for the recovery of cocoa polyphenols was used. The extraction of unfermented cocoa beans after the polyphenol oxidase enzyme inactivation, followed by ultrasound-assisted solid–liquid extraction, resulted in a yield of 168 mg of crude cocoa extract per g cocoa beans (dry matter basis). The total polyphenol and total flavonoid were 122.3 mg gallic acid equivalent per gram and 88.6 mg (–)-Epicatechin equivalent per gram, respectively. In fact, our extract was composed by 65 wt.% procyanidins followed by 20 wt.% alkaloids and 15 wt.% catechins; the major alkaloid was theobromine (7.8 mg/g) for a theobromine/caffeine ratio equal to 2.9, and 7.3 mg of (–)-Epicatechin per g of cocoa bean, which was 11.8-fold higher than (+)-Catechin. Regarding oligomeric PCs, the major procyanidin was the Trimer C1 (11.9 mg/g) being 2.9-, 1.3-, 1.7-, 6.6-fold higher than the dimer, tetramer, pentamer, and hexamer, respectively [49]. 

Furthermore, different techniques to fractionate procyanidins from cocoa polyphenolic extract were evaluated and compared. As mentioned, classical fractionation techniques based on solid phase extraction and gel permeation column were chosen for being straightforward and not requiring special equipment, and preparative HPLC with two different polarity columns was also studied. 

### 2.1. Separation Using Solid Phase Extraction

**Figure 1:**
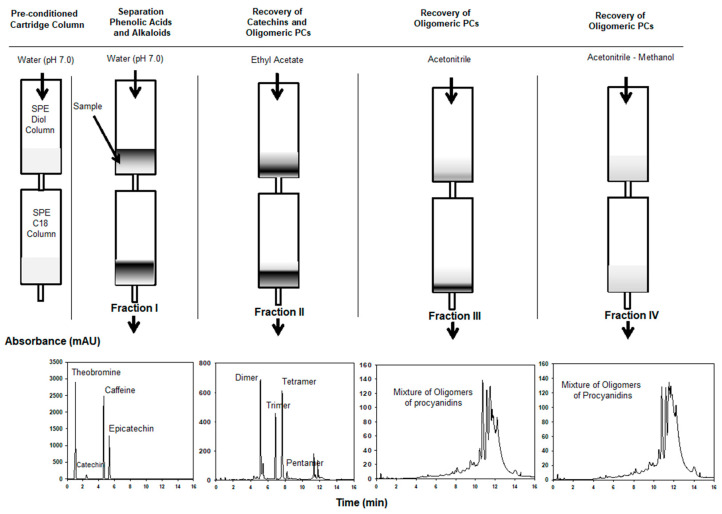
Scheme for the fractionation of procyanidins using SPE cartridges diol-C18 coupled in series.

In previous assays, the individual evaluation of C18 and diol cartridges was ineffective to separate procyanidins as well as catechins. For example, the fractions obtained by C18 consisted in typical hump of oligomeric procyanidins, while fractions enriched with mixture of procyanidins were enhanced by diol cartridge. Better results were obtained when diol and C18 cartridges were coupled in series (Figure 1) due to the orthogonality of the system. Therefore, the solvents chosen to elute the sample were based on the like-attracts-like principle. As the main interest is the isolation of procyanidins, the first step was to remove the alkaloids, phenolic acids, and catechins; therefore, water (pH 7) was chosen for this purpose. Then, separation of the oligomeric procyanidins was done according to their solubility within the solvent: first, ethyl-acetate (non-polar and slightly polar solvent) was used, followed by acetonitrile and acetonitrile–methanol to favor elution of high polar compounds. In addition, to ensure the reproducibility of this technique, several load concentrations (1–50 mg) of cocoa extract were assayed. At low load, high reproducibility, short separation time (<20 min, 2 drops/s), and low separation yield were achieved, while, at high load (≥20 mg), the cocoa extract clogged the solid-phase media and resulted in extremely slow sample extraction (>1 h). Therefore, a load equal to 15 mg cocoa extract was chosen. Four fractions were collected and analyzed by UHPLC-QTOF-MS. Fraction I consisted mainly of theobromine, caffeine, (‒)-Epicatechin, and a small amount of (+)-Catechin (yield = 0.19%), while Fractions II–IV were enriched with DP of 2–5, 5–8, and 4–9, and yields equal to 0.18%, 0.9%, and 1.2%, respectively (Figure 1). These data confirmed that SPE (solid phase extraction) separation was not selective for extracting pure procyanidins that are very diverse in structure within the same molecular weight. 

### 2.2. Separation on Sephadex LH-20

**Figure 2:**
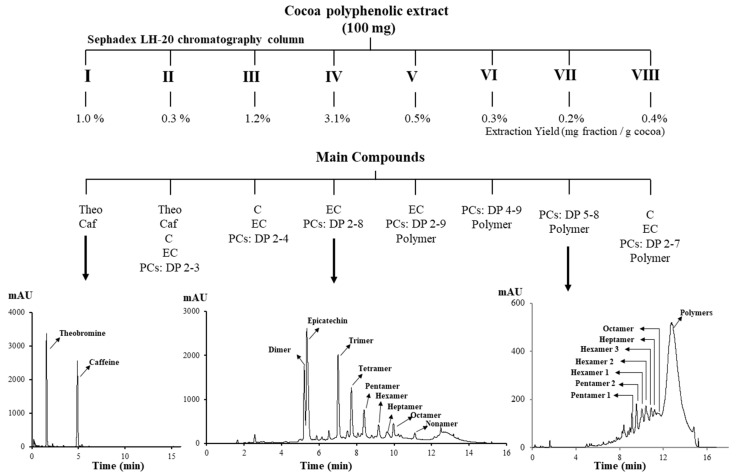
Scheme for the separation of cocoa procyanidins using Sephadex LH-20. Theobromine, Theo; Caffeine, Caf; (+)-Catechin, C; (–)-Epicatechin, EC; PCs, procyanidins; DP, degree of polymerization.

Sephadex LH-20 was chosen as a second classical fractionation technique for being simple and widely reported for the separation of non-polymeric [50] procyanidins [51] and polymeric procyanidins [4,35] but it is time consuming (ca. 2 h). Following the procedure by Kennedy and Taylor (2003) [52], eight fractions were collected (see Section 4.3.2. for fractions’ elution); their main constituents are detailed in Figure 2. The chromatographic profiling at 280 nm showed that Fraction I consisted mainly of theobromine and caffeine (yield = 1.0%), whereas Fractions II and III (yield equal to 0.3% and 1.2%, respectively) contained catechins, traces of oligomeric PCs (DP 2–4), and alkaloids. In fact, these fractions were obtained using aqueous methanol, which enables the recovery of non-phenolic compounds and PCs with DP ≤ 4. A subsequent elution by increasing the concentration of acetone in methanol allowed the recovery of oligomeric PCs (Fractions IV–VII), but all the fractions consisted in a mixture of PCs with different DP and a hump approximately at 12.5 min of retention time corresponding to co-eluted polymeric polyphenols; moreover, differences among yields (0.2–3.1%) were observed (Figure 2). The last fraction, corresponding to the elution with acetone–water–methanol, showed a lower DP than previous fractions since washing phase could dissolve low molecular procyanidins adsorbed on the stationary phase. In agreement with our results, Neto et al. [53] obtained the fractionation of procyanidins from cranberry, being able to separate eight fractions consisting of a mixture of oligomeric procyanidins (DP 2–11). These results suggest that individual PC compounds cannot be separated using classical gel permeation column.

### 2.3. Separation by Preparative HPLC Under Reversed Phase Conditions

To achieve successful separation of procyanidins using a preparative C18 column, run time, solvent gradient, and flow rate were optimized. In fact, analysis of procyanidins at analytical scale employing C18 columns has been previously assayed [54]. However, the scale up from analytical scale to preparative scale is still a challenge. Therefore, different factors influencing the final separation were tested: column temperature of 25–55 °C, flow rate from 8 to 30 mL/min, and gradient that was modified by changing the percentage of ethanol (Solvent B) (increasing 5% B in each test) as well as the amount of acetic acid (0.1–2.0% *v*/*v*) in Solvent A.

In general, lower flow rates increased analysis time and improved neither the separation nor procyanidins peak shape. On the other hand, at faster flow rate, procyanidins with higher molecular weights eluted as an unresolved peak, because of the high linear velocity of the mobile phase that decreases the possibility of interaction between the analytes and the stationary phase. 

No significant changes (*p* < 0.05) on procyanidin separation were observed with: (a) increasing the concentration of acid present in Solvent A and, therefore, the lowest concentration of acid was chosen; and (b) cocoa extract concentration of 70–85 mg/load. As expected, increasing the temperature to 50 °C resulted in the improvement of resolution of dimers, trimers, and tetramers but was inefficient to completely separate higher oligomers and polymers; e.g., fractions consisting on DP > 5 co-elute with oligomeric procyanidins and led to poor separation (Table 1). Overall, the best separation conditions consisted of acidified water (0.1% acetic acid, Solvent A) and ethanol (Solvent B) at 55 °C and a flow rate of 12 mL/min (more details in methodology, Section 4.3.3). 

**Figure 3:**
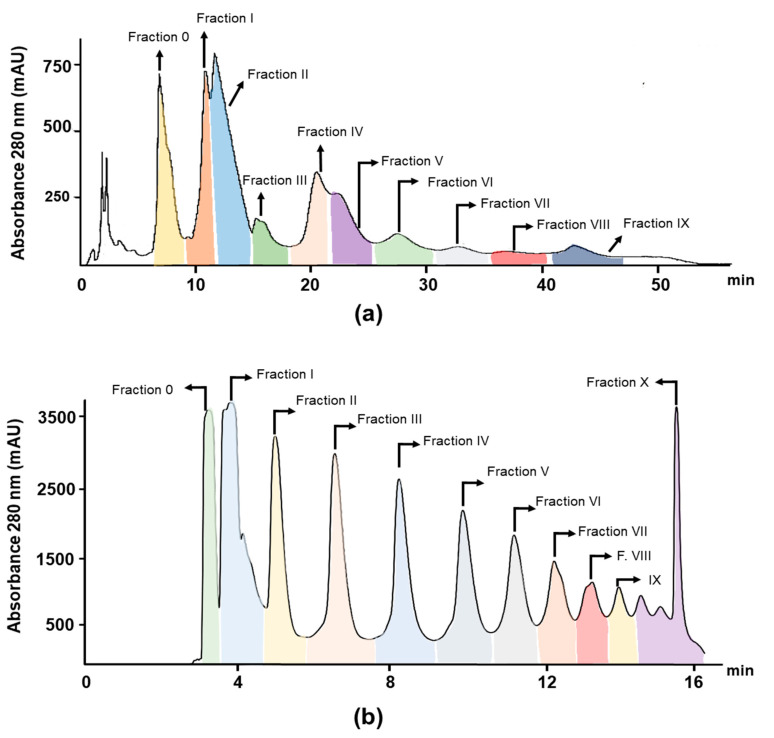
Preparative chromatogram obtained at optimum conditions using: (**a**) C18 stationary phase; and (**b**) diol stationary phase columns.

As shown in Figure 3a and Table 1, the elution order of cocoa extract consisted on a first elution of theobromine (6.8 min), followed by dimer (10.8 min), (–)-Epicatechin (11.8 min), trimer (20.4 min), and mixtures of higher oligomers (DP 4–7) from 22.2 to 42.8 min. Table 1 summarizes the tentative identification of the main compounds collected using reversed phase, as well as their main MS and MS/MS features. For instance, several fractions were collected showing relative composition >95% for theobromine (fraction 0), caffeine (Fraction III), and (–)-Epicatechin (Fraction II). As we previously reported, good separation was observed for Fractions I (ca. 95% dimer B2 and 2% (–)-Epicatechin) and IV (ca. 3% dimers B2, 80% trimer C1, 10% tetramers, and 7% pentamers) [55]. For other fractions, it was difficult to obtain purities around 80% and, as can be observed in Table 1, they were composed of mixtures of different oligomers, e.g. Fraction V consisted of 3% dimers, 10% trimers, 54% tetramers, and 33% pentamers. Despite the careful optimization, the complexity of the sample and the number of isomers of procyanidins, which imply a mixture of very similar chemical structures closely eluting, did not allow a complete separation of oligomers (Figure 3a).

### 2.4. Separation by Preparative HPLC Using a Diol Column

The selection of mobile phase and column was according to Kelm et al. [48]. The optimization of the separation of procyanidins was aimed at reducing solvent consumption while improving method reproducibility. As the separation occurs by increasing degrees of polymerization, the concentration of Solvent B plays a key role on the complete separation of each procyanidin. Thereby, several experiments were performed to obtain the optimum gradient. For instance, it was observed that starting at 8–10% Solvent B (methanol/water/acetic acid, 95/3/2 *v*/*v*/*v*) allowed a fast separation of alkaloids and monomeric catechins; however, when higher initial amount of Solvent B was tested, a co-elution of oligomers and loss of baseline separation was obtained. Finally, a good separation of oligomers was achieved with a slow gradient of Solvent B. Moreover, complete elution of polymeric PCs was reached using 100% of Solvent B in the mobile phase. 

Once the gradient was optimized, the target was to reduce analysis time, minimize pressure changes, and achieve better resolution among high molecular weight procyanidins. The first factor to optimize was the column temperature within 30–50 °C. As expected, faster molecular diffusion and better separations were obtained at higher temperature (50 °C). The effect of sample solvent was tested with the objective of dissolving PCs while, at the same time, removing non-phenolic compounds. Indeed, Kelm et al. [48] reported that the solubility of the cacao procyanidin extract in the mobile phase limits the amount of material in one injection; therefore, sample preparation to increase the injection load/injection was studied. Therefore, raw cocoa extract was dissolved in Solvent A (acetone/water/acetic acid, 70/29.5/0.5, *v*/*v*/*v*), Solvent B (methanol/acetonitrile/acetic acid, 38/60/2, *v*/*v*/*v*), and Solvent C (acetone/water/acetonitrile/acetic acid, 60/29.5/10/0.5, *v*/*v*/*v*/*v*). After dissolution, the sample was centrifuged (5000 *g*, 5 min) and the supernatant was collected, filtered, and injected into the system. The results show better resolution of procyanidins with Solvent A, but it caused an increase in the column backpressure because of the progressive precipitation of sample with the mobile phase and higher contamination of the column. Using Solvent B, higher shift on retention times and higher distortion of the peaks was observed. Therefore, Solvent C was selected since it allowed elimination of interfering compounds without compromising the separation and the performance of the column and the chromatographic system. 

Sample loading from 30 to 150 mg per injection was also investigated. As expected, higher amounts (>100 mg) led to loss of baseline, co-elution of PCs, low resolution, and system overpressure, whereas a small amount decreased the overall yield of the process. As a result, 70–85 mg of sample was selected as the optimum range for a good balance among yield, time, and reproducibility of the process. Moreover, to enhance separation performance, it was necessary to reduce the internal capillary tubing and the flow path in the chromatographic system. Thus, 0.17 mm ID capillary tubing and short capillary length, which contributed to decrease the friction along the walls and avoid the extra-column band broadening, were selected. 

In summary, fractionation of procyanidins using a preparative diol column was achieved at 5 mL/min and 50 °C column temperature using the following linear gradient: 0 min, 10% B; 0.5 min, 12% B; 1.5 min, 12% B; 6.0 min, 18% B; 12.5 min, 35% B; 12.6 min, 100% B; 13.6 min, 100% B; 13.7 min, 10% B; 23.7 min, 10% B. 

A similar work was proposed by Kelm et al. [48], who could separate up to heptameric procyanidins in a diol-column but run time analysis was longer (ca. 70 min) using a high flow rate of 55 mL/min. Thereby, the improved method not only enhanced resolution of oligomeric procyanidins (DP ≤ 14) but also the speed of separation (18 min), making this technique more cost effective. In addition, a complete characterization of the isolated fractions and their main isomeric forms could be done in the present work. Moreover, a study was performed on how to concentrate the sample without affecting their structure and composition, as explained below. 

As shown in Figure 3b, good separation of the different procyanidins by increasing degree of polymerization was achieved. Fractions 0–X were collected, pooled, and used for further experiments. Pooled fractions were concentrated following three procedures: (P_1_) rotary evaporation at 30 °C under vacuum plus drying with N_2_ at 25 °C; (P_2_) neutralization of each fraction using 0.1N NaOH plus drying with N_2_ at 25 °C; and (P_3_) drying using N_2_. 

The results show that, during the rotary evaporation, the concentration of acid increased and led to degradation or oxidation of the sample. Regarding treatment P_2_, the formation of salt as a result of the reaction between NaOH and CH_3_COOH was observed and, as a consequence, interfering compounds were detected during the analysis. Treatment P_3_ was carried out successfully without affecting the chemical structure of the present compounds. 

**Figure 4:**
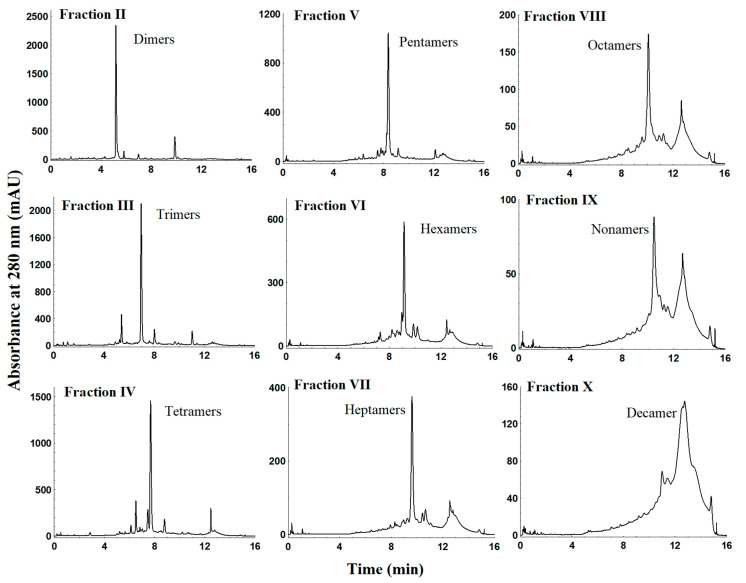
UHPLC profiles of oligomeric fractions obtained using a diol column.

**Figure 5:**
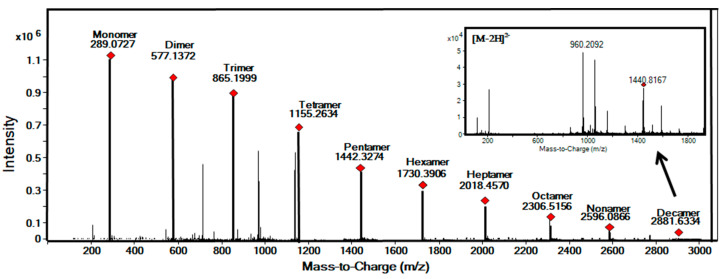
Negative ESI-QTOF average mass spectrum obtained for all the cocoa procyanidins fractions.

Chromatograms recorded at UV 280 nm for Fractions II–X and average mass spectrum corresponding to those fractions are shown in Figure 4 and Figure 5, respectively. As shown in Table 2, Fractions II–VI showed [M-H]^–^ ions at *m*/*z* 577.1358, 865.1993, 1153.2624, 1441.3170, and 1729.8774, corresponding to dimers, trimers, tetramers, pentamers, and hexamers, respectively. As expected, all collected fractions consisted exclusively of isomeric PCs with the same DP; for instance, Fraction III presented 16 main peaks, all of them identified as trimers, showing [M − H]^−^ ions at *m*/*z* 865.1993 and MS/MS fragmentation patterns including ions at *m*/*z* 575.1205, 287.0566, and 125.0241. In addition, doubly charged ions ([M − 2H]^2–^) detected at *m*/*z* 1008.7263, 1152.7555, 1296.7871, and 1440.8102 that corresponded to heptamer, octamer, nonamer, and decamer structures, respectively, were also identified in Fractions VII–X.

These finding confirmed the high diversity of chemical structures of procyanidins. Indeed, considering only molecules with Catechin and Epicatechin, the number of asymmetric carbon atoms and the number of possible interflavan bonds (C_4_–C_8_ or C_4_–C_6_), theoretically, for higher oligomer and polymers, the possible number of stereoisomers is $2^{\left(4*DP-2\right)}$ [56]. For example, 64, 1024, and 16,384 combinations for dimers, trimers, and tetramers could be found, respectively.

### 2.5. Antioxidant Activity of Cocoa Extracts and Its Procyanidins Fractions 

**Figure 6:**
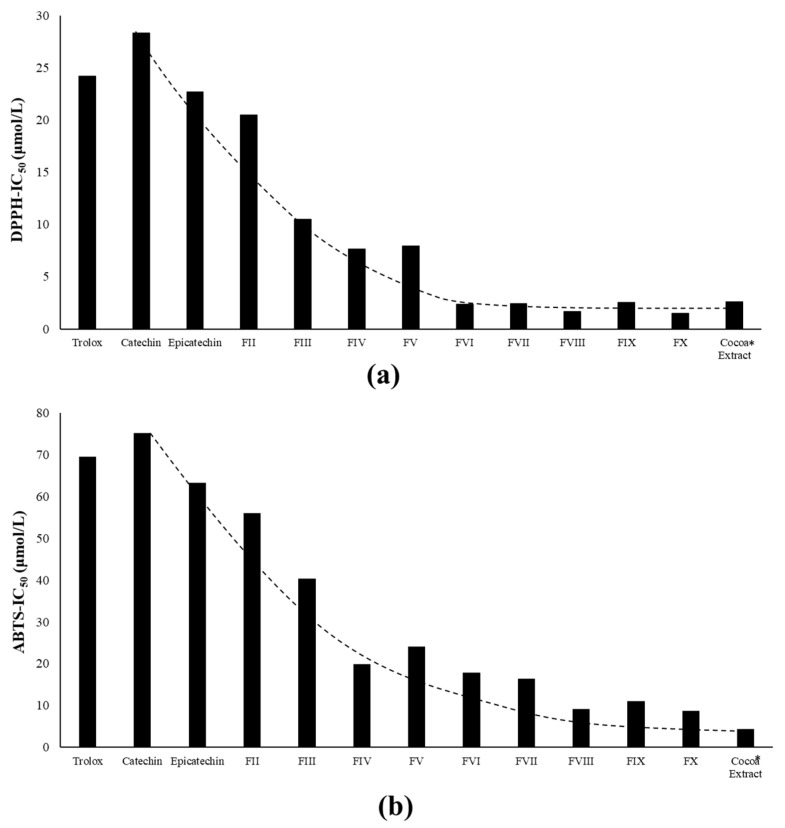
Antioxidant activity of cocoa extract and its procyanidins fractions by: (**a**) DPPH^•^-IC_50_; and (**b**) and ABTS^•+^-IC_50_. Fractions II–X refer to procyanidin separation by diol-column phase (see Table 2). Cocoa extract is expressed as (–)-Epicatechin equivalents. The dotted line represents the scavenging activity trend. Procyanidins’ antioxidant activity displayed exponential behavior by increasing the degree of polymerization ranging from monomer to oligomers (up to DP ≤ 7) and slight increase for oligomers (DP ≥ 7) up to polymers.

The IC_50_ value was determined using both DPPH^•^ and ABTS^•+^ for free radical scavenging activity assays. In general, the kinetics of the reaction to inhibit DPPH^•^ or ABTS^•+^ radicals were dependent on the concentration of tested compounds (data not shown). Furthermore, the free radical scavenging activity was chemical structure-dependent, as shown in Figure 6.

The IC_50_ values determined by the DPPH^•^ assay were significantly higher than those of the ABTS^•+^ test in all tested samples. The difference between antioxidant activities could be related with the higher solubility of samples in methanol than PBS solution. In fact, the ABTS^•+^ assay is based on the generation of a blue/green ABTS^•+^, which is applicable to both hydrophilic and lipophilic antioxidant systems, whereas the DPPH^•^ assay uses a radical dissolved in organic media, therefore applicable to hydrophobic systems [57].

Cocoa procyanidins fractions showed the ability to prevent radical formation, as shown in Figure 6. In fact, DPPH^•^ and ABTS^•+^ were positively correlated to PC degree of polymerization; therefore, highest antioxidant activity was in the following order: FX > FIX > FVIII > FVI I > FVI > FV > FIV > FIII > FII > (–)-Epicatechin > Trolox > (+)-Catechin. As explained by Pedan et al. [58], from the chemical point of view, this seems to be reasonable, as those large molecules provide more structural features for interacting with radicals. 

In accordance with previous authors, Arteel and Sies [59] found that long-chain PCs are better against the nitration reaction than short-chain PCs; in particular, tetramer was the most efficient inhibitor. In a preparative high-speed counter-current chromatography separation of grape seed procyanidins, similar orders for both DPPH-IC_50_ and ABTS^•+^-IC_50_ were found [56]. Polymeric PCs showed hereby to be more efficient free radical scavengers than oligomeric and monomeric fractions. These results also underline the observations by Spranger et al. [60], who found that high DPPH^•^ scavenging activity was in the following order: polymers > oligomeric >> Trolox ≈ ascorbic acid ≈ (+)-Catechin. In fact, Braunlich et al. [61] found that DPPH^•^-IC_50_ for procyanidin trimer C1 was 1.45-fold higher than dimer B2. 

Regardless, cocoa crude extract (expressed as (–)-Epicatechin equivalents) was proven to be an efficient free radical scavenger despite its high molecular weight and potential steric hindrance. For example, cocoa extract was 11.3- and 17.3-fold more efficient than (+)-Catechin for DPPH^•^-IC_50_ and ABTS^•+^-IC_50_, respectively.

## 3. Discussion 

Considering the available literature, this study reinforces the advantages of preparative HPLC compared to classical fractionation methods such as gel permeation or solid phase cartridges. In general, diol-C18 cartridges coupled in series and Sephadex LH-20 columns provide simple methods for the separation of non-phenolic and phenolic compounds. However, no isolation of individual components can be achieved. In fact, the relative composition of the individual procyanidins was poor; for instance, the relative composition of each procyanidin was 33.3% (dimer), 22.2% (trimer), 37.0% (tetramer), 0.3% (pentamer), and 7.2% (polymer) in Fraction II obtained by diol-C18 cartridges, and 14.3%, 33.9%, 19.8%, 13.1%, 8.2%, 3.9%, 3.0%, and 3.8% from monomeric to octameric PCs in Fraction IV obtained by Sephadex LH-20. These results are in agreement with those reported by Pedan et al. [62] who found that complex groups, such as polymeric PCs, interacted irreversibly with Sephadex LH-20. This can be due to the separation mode, which is mainly based on adsorption phenomena rather than size-exclusion and, thus, polyphenols containing PCs are partly separated according to the different affinity for the gel matrices [37]. It can be concluded that classical techniques are mainly recommended for the isolation of alkaloids, a mixture of PCs with DP < 3 and a mixture of high molecular weight oligomeric compounds. 

According to the mass spectrometry-based characterization, better separation, and a limited number of isomers of procyanidins are obtained by preparative HPLC using a C18 column as a stationary phase, as shown in Table 1 and Figure 3. The results show that preparative reverse phase was effective for separation of individual alkaloids, catechins, and small oligomeric weight procyanidins, in particular, dimer B2, and trimer C1. Indeed, latter fractions’ yields reinforced that theobromine and (–)-Epicatechin are the most abundant compounds in cocoa extract followed by dimer and trimer fractions with yields equal to 0.44% and 0.58%, respectively. Fractions V–IX were composed of several procyanidins with different degree of polymerization. In addition, an increase of backpressure over time was also observed and, therefore, a clean-up procedure between batches was needed. This phenomenon could be a result of the adsorption of higher oligomers onto the stationary phase. One of the advantages of this procedure was the use of ethanol as mobile phase, which makes this process cheap and free of toxic solvents. The main drawback was that it required a small injection volume to allow high reproducibility, thus providing low yields of the target compounds.

On the other hand, semi-preparative diol-HPLC afforded higher reproducibility, higher yield, and higher separation power of procyanidins (both oligomeric and polymeric) compared to the classical separation and silica phase. In fact, using HILIC method (processing 1 g cocoa extract), the total of fractionating and solvent consumption was ca. 5 h and 1.5 L, i.e., 75.5% and 93.0%, 77.9% and 70.5%, and 60.8% and 83.6% lower than with gel permeation, solid phase separation, and RP-LC, respectively. 

As shown in Table 1 and Table 2, C18 and diol column displayed similar yields for alkaloids (1.32% vs. 1.26%), but diol-column’ yield was slightly greater for monomeric (0.92% vs. 1.41%) and oligomeric procyanidins DP ≥ 2 (2.50% vs. 4.79% total sum), which is due to the ability of diol to separate by molecular weight procyanidins and the isolated chromatographic peak was composed of several isomeric forms. For example, trimer fraction was composed of four isomers by C18 column and low-resolution separation together with coelution of tetramers and pentamers (Figure 3a, Fraction IV), while by diol-column trimer peak was mainly composed of 16 isomers and low amount of (–)-Epicatechin and dimer (Table 2). Nevertheless, diol-column separates based on DP, thus pure procyanidins cannot be isolated. To overcome this problem, the combination of both mechanisms (diol and reverse phase) deserves further research. In this way, we recently reported an analytical comprehensive online LC×LC (HILIC × reverse phases) method for the separation of cocoa procyanidins [63], being able to separate 24 isomeric forms, and the preparative separation is currently underway.

Regarding the antioxidant activity, this study has proven that antioxidant activity increases linearly for monomer to oligomers but increases slight for polymers (DP > 7), which could be explained by the dependence of polymer molecular weight and hydrogen electron donating effect. In general, cocoa extract and fractions consisting of octamers to decamers showed the highest free radical scavenging activity related to monomeric compounds and low molecular weight oligomers (Figure 6a,b). Therefore, it was demonstrated that high effectiveness against radicals is enhanced by cocoa procyanidins and extends to higher molecular weight. This study highlights the antioxidant potential of oligomeric procyanidins; in fact, Ramos et al. [64] showed that anti-diabetic effects of cocoa extract in cultured cells is related to the content and the degree of polymerization of cocoa procyanidins. 

## 4. Materials and Methods

### 4.1. Reagents and Standards

All chemicals used were of analytical or reagent grade and were not purified further. (+)-Catechin hydrate (≥99%; ASB-000003310), (–)-Epicatechin (≥99%; ASB-00005127), and procyanidin B1 (≥90%; ASB-00016230) were purchased from ChromaDex Inc. (Irvine, CA, USA). Procyanidin dimer B2 ((–)-Epicatechin (4β-8)-(–)-Epicatechin) and trimer C1 ((–)-Epicatechin (4β-8)-(–)-Epicatechin (4β-8)-(–)-Epicatechin) were obtained from Cymit quimica (Barcelona, Spain). Theobromine, caffeine, formic acid, sodium hydroxide, glacial acetic acid, 2-diphenyl-1-picrylhydrazyl (DPPH), 2,2’-azino-bis (3-ethylbenzothiazoline-6-sulfonic acid) diammonium salt (ABTS), potassium persulfate, phosphate-buffered saline (pH 7.4), and Trolox ((±)-6-hydroxy-2,5,7,8-tetramethylchromane-2-carboxylic acid), were obtained from Sigma Aldrich (St. Louis, MO, USA). Acetonitrile, methanol, and acetone were of HPLC-grade and acquired from VWR Chemicals (Barcelona, Spain). HPLC-grade ethanol was obtained from Fisher Scientific (Fair Lawn, NJ, USA). Sephadex LH-20 was obtained from GE Healthcare (GE Healthcare GmbH, Freiburg, Germany). Milli-Q water (Millipore system, Billerica, MA, USA) was used for the preparation of all solutions. 

### 4.2. Food-Safe Process for Ultrasound-Assisted Solid–Liquid Extraction of Cocoa Procyanidins 

Cocoa polyphenolic extract was produced on a lab scale from unfermented cocoa beans (Trinitary variety ICS-39 were clone in Colombia) using the procedure described by Toro-Uribe et al. [49,55]. Briefly, the unfermented cocoa beans (pulp free) with low polyphenol oxidase enzyme activity was obtained by placing the beans in a solution of 70 mM ascorbic acid/L-cysteine (1:1 *v*/*v*) at 96 °C for 6.4 min followed by immediately cooling in ice water for 30 min according to Toro-Uribe et al. [65]. Then, the beans were oven dried at 70 °C for 3 h (Binder model FD 23, Tuttlingen, Germany) and ground using a cryogenic ball mill (Cryomill MM 400, Retsch GmbH, Haan, Germany). The resulting powder was used for an ultrasound-assisted solid–liquid extraction using aqueous ethanol; thus, cocoa powder (1 g) was mixed with 120 mL of 50% ethanolic solution and ultrasonicated at 35 kHz for 30 min (Elma, Ultrasonic LC20H, Singen, Germany), followed by incubation at 70 °C for 45 min under constant stirring. The resulting extract was centrifuged (5000 *g*, 4 °C, 20 min) (Heraeus, Megafuge 16R, Thermo Scientific, Waltham, MA, USA) [49,55]. The supernatant was recovered and concentrated until a resulting violet-powder was obtained. The total polyphenolic and total flavonoid contents were determined according to Singleton et al. [66] and Zhishen et al. [67], respectively. 

### 4.3. Fractionation of Procyanidins 

#### 4.3.1. Solid Phase Separation Using Diol-C18 Cartridges Connected in Series

Solid phase extraction was performed with SPE-Cartridge C18 (300 mg; Lida, Kenosha, WI, USA) and SPE-Cartridge diol (500 mg, Discovery DSC-diol, 70-Å pore diameter, Supelco, Bellefonte, PA, USA). The diol–C18 cartridges were connected in series. Cartridges were preconditioned with 20 mL water (pH 7.0). Then, the sample was allowed to be adsorbed into the matrix by gravity. After that, the different solvent mixtures were sequentially passed through by negative pressure (vacuum manifold instrument operated at 0.03 MPa) as follows: 10 mL water (pH 7, Eluent I) to flush out the phenolic acids; 15 mL ethyl acetate (Eluent II) to elute alkaloids, catechins and low molecular weight PCs; 15 mL of acetonitrile (Eluent III); and then 15 mL of acetonitrile–methanol (60/40 *v*/*v*). The preparation of the cocoa extract consisted of dissolution in water with 0.01% formic and filtered by hydrophilic Durapore PVDF membrane 0.45 µm (Millipore, Bedford, MA, USA) for a final load equal to 15 mg. 

The concentration of the fraction was carried out under a gentle flow of nitrogen gas at 30 °C (TurboVap LV, Caliper Life Science, Hopkinton, MA, USA). Then, the dried fractions were stored at −80 °C until further analysis. All fractions were analyzed by UHPLC-QTOF-MS method described in Section 4.4 to determine its final composition.

#### 4.3.2. Column Chromatography by Sephadex LH-20

Ten grams of Sephadex LH-20 were swelled in excess of methanol, and stirred (Thermomixer, Eppendorf, Hamburg, Germany) at room temperature for 6 h. Then, a glass column (3 cm × 50 cm), plugged with cotton wool and a layer of sand (0.6 cm), to prevent the leaking of the stationary phase, was first washed with methanol and then carefully packed with a slurry containing Sephadex LH-20 in methanol. To ensure complete packing, vacuum (0.08 MPa) was applied by attaching the vacuum tubing to the bottom of the column without deforming the bed. After that, the column was equilibrated with five-bed volumes of 60% (*v*/*v*) aqueous methanol at a flow rate of ~6 cm/h. The cocoa extract (100 mg) was dissolved in methanol–water–formic acid (60/39.9/0.1 *v*/*v*/*v*), filtered by hydrophilic Durapore PVDF membrane 0.45 µm (Millipore, USA), and applied to the Sephadex LH-20 column. The solvent system for the separation of PCs was based on Kennedy and Taylor [52] with few modifications as follows: 60% (*v*/*v*) aqueous methanol (Eluent I), 75% (*v*/*v*) aqueous methanol (Eluent II), 90% (*v*/*v*) aqueous methanol (Eluent III), methanol–water–acetone (80/10/10 *v*/*v*/*v*) (Eluent IV), methanol–water–acetone (65/15:20 *v*/*v*/*v*) (Eluent V), methanol–water–acetone (40/30/30 *v*/*v*/*v*) (Eluent VI), 60% (*v*/*v*) aqueous acetone (Eluent VII), and methanol–water–acetone (30/50/20 *v*/*v*/*v*) (Eluent VIII). Each eluent (consisting on 200 mL) was applied sequentially, and the corresponding pooled fractions were immediately concentrated as above mentioned (Section 4.3.1). All fractions were analyzed by the UHPLC-QTOF-MS method described in Section 4.4 to determine its final composition.

#### 4.3.3. Preparative Isolation by Reverse Phase HPLC

As we previously reported [55], the low-Pressure Gradient Preparative System HPLC (Prominence LC, Shimadzu, Columbia, MD, USA) consisting of a degasser, quaternary pump (LC-20AP), UV–Vis detector, autosampler, and automatic fraction collector (FCV-200AL) was operated at 12 mL/min using as solvents acidified water (0.1% acetic acid, Solvent A), and pure ethanol (Solvent B). The optimal linear gradient was: 0 min, 6% B; 20 min, 10% B; 30 min, 15% B; 40 min, 30% B. After each injection, the column reached 80% B for 5 min followed by 15 min re-equilibrations of the column to initial conditions. The preparative HPLC column was a C18 (250 mm × 10 mm, 10 µm, Alltech, Deerfield, IL, USA) connected to guard column refill C18 (Part # 28551, Alltech, Deerfield, IL, USA) and was operated at 55 °C using a temperature control module (Model TC2, Millipore Waters, Marlboro, USA).

The preparation of the sample consisted of dissolution in Solvent A and then filtering through 10-µm and then 2-µm pore size filters (nylon membrane filter, diameter 47 mm, GVS Maine Magna, USA). Each injection volume was equivalent to 80 mg/load. Isolated fractions were initially stored at −20 °C and concentrated using a vacuum evaporator connected to a dry-ice cold trap (R-114, Büchi, Flawil, Switzerland) followed by freeze-drying in a VirTi Genesis freeze dryer (SP Inc, Gardiner, NY, USA) for 72 h and finally stored at −80 °C under nitrogen atmosphere until further analysis. The relative composition of each fraction was determined by the UHPLC-QTOF-MS method described in Section 4.4. 

#### 4.3.4. Preparative Isolation by HPLC Using a Diol Column

A semi-preparative HPLC instrument (1290 infinity series II, Agilent Tech., Santa Clara, CA, USA) consisting of a degasser, quaternary pump, UV–Vis detector, thermostated autosampler, and oven, equipped with an automatic fraction collector, was used. The separation was performed on a Develosil diol column (250 mm × 10 mm, 5 μm) at 50 °C. The flow rate was set at 5 mL/min, and the injection volume was equivalent to 80 mg/load. The mobile phase was based on a previous work by Kelm et al. [48], using acetonitrile/acetic acid (98/2 *v*/*v*, A) and methanol/water/acetic acid (95/3/2 *v*/*v*, B). The optimal linear gradient applied was: 0 min, 10% B; 0.5 min, 12% B; 1.5 min, 12% B; 6.0 min, 18% B; 12.5 min, 35% B; 12.6 min, 100% B; 13.6 min, 100% B. Then, it was returned to the initial condition (10% B) and re-equilibrated for 10 min. The sample was dissolved in acetone/water/acetonitrile/acetic acid (60/29.5/10/0.5 *v*/*v*/*v*/*v*) and filtered through a 0.45-µm (Durapore PVDF, Millipore, USA) membrane. The thawed fractions were pooled and stored at −80 °C for less than 5 days. After that, collected samples were concentrated and stored as explained in Section 4.3.1. The relative composition of every fraction was determined by UHPLC-QTOF-MS method described in Section 4.4.

### 4.4. Characterization by UHPLC-QTOF-MS

Analyses were performed with an Agilent UHPLC (1290 Infinity series, Agilent Tech., Santa Clara, CA, USA), consisting of a degasser, binary pump, column oven, UV–Vis detector and thermostated autosampler. The data acquisition was carried out using Mass-Hunter LC/MS software (version 5.01, Agilent). All samples were dissolved in water with 0.01% formic acid, and 4 µL were injected into a C18 reverse phase Zorbax Eclipse Plus column (50 mm × 2.1 mm, 1.8 µm) connected to a Zorbax SB-C8 guard column (5 mm × 2.1 mm, 1.8 µm) maintained at 55 °C. The flow rate was 0.7 mL/min with Solvent A composed of water with 0.01% formic acid, and organic Solvent B composed of acetonitrile with 0.01% formic acid in negative ionization mode. For positive ionization mode, the concentration of formic acid was 0.1% in both Solvents A and B. Gradient elution was applied as follows: 0 min, 0% B, 3.9 min, 1.5% B; 4.0 min, 4% B; 11.0 min, 10% B, 14.0 min, 35% B; 14.2 min, 100% B; 16.5 min, 100% B; 17.0 min, 0% B; 23 min, 0% B. The resulting separation was recorded at 280 nm. All samples were injected by triplicate. Additionally, a blank sample was injected between every sample. The composition of isolated fractions was carried out using analytical standards (theobromine, caffeine, (–)-Epicatechin, procyanidins Dimer B1, Dimer B2, and Trimer C1). Further elucidation of high oligomeric procyanidins was carried out by MS due to the lack of commercial standards.

The data were collected in negative ESI mode and selected samples were also analyzed in positive ESI mode on a QTOF-MS instrument (model 6540, Agilent, Waldbronn, Germany) operated in full scan mode over *m*/*z* 25–3200 using the following settings: capillary voltage, 4000 V; acquisition rate, 2 spectra per second; nebulizer pressure, 40 psi; drying gas, 10 L/min; and temperature, 350 °C. During the analysis, two reference masses (C_5_H_4_N_4_ and C_18_H_19_O_6_N_3_P_3_F_24_) were used. Thus, *m*/*z* 119.0363 and *m*/*z* 966.0007 for negative and *m*/*z* 121.0508 and *m*/*z* 922.0097 for positive mode were employed. These masses were continuously infused to the system to allow constant mass correction. Data treatment was performed using Mass-Hunter Qualitative Analysis (Agilent, B.07.00). MS characterization features were analyzed using commercial standards reagents, extraction ion compound tool, and exact mass databases searched against the METLIN and HMBD databases. 

Relative composition was only enhanced for the fractions consisted on theobromine, caffeine, (+)-Catechin, (–)-Epicatechin, dimer B1, dimer B2, and trimer C1. Due to the lack of commercial standard for oligomeric procyanidins, their relative composition in percentage of individual compounds was calculated as follows:

$$Relative\;Composition_{i}\left(\%\right)=\frac{Area_{Compound_{i}}}{\sum_{i}^{k}Area_{Compound}}*100$$

where *i* is the individual isomeric procyanidin and $\overset{k}{\underset{i}{\sum }}Compound_{Area}$ is the total sum of the areas from all procyanidins presented in the fraction. 

The separation yield was calculated as follows:

$$Yield\;\left(\%\right)=\frac{weight\;fraction\;\left(mg\right)}{weight\;cocoa\;extract\;\left(mg\right)\;}*100$$

### 4.5. Antioxidant Activity Assays

The activity of cocoa extract and its procyanidins fractions to stabilize the formation of DPPH^•^ and ABTS^•+^ radicals were also assayed. The (+)-Catechin, (–)-Epicatechin, cocoa procyanidins fractions and Trolox were in the concentration range of 0–500 µM. For cocoa crude extract, the concentration range was 0–250 ppm, and expressed as (–)-Epicatechin equivalent as follows:

$$Cocoa\;extract\;\left(\frac{\mu mol\;ECE}{L}\right)=\frac{weight_{cocoa\;extrac}\;\left(mg\right)}{L}*Y*C_{EC}*MW_{EC}\;$$

where *ECE* is the (–)-Epicatechin equivalent, *Y* is the extraction yield equal to 168 mg of crude cocoa extract/g cocoa beans (dry matter basis) [49], $C_{EC}$ is the concentration of (–)-Epicatechin equal of 7.3 mg EC/g cocoa bean [49], and MW is the molecular weight of (–)-Epicatechin.

DPPH^•^ assay was carried according to procedure by Brand-Willians [68] with the following modifications: 27.4 mM DPPH^•^ methanolic solution was diluted with methanol to obtain an absorbance of 0.57 ± 0.01 units at 517 nm using a microplate reader (Synergy HT, BioTek Instruments, Winooski, VT, USA) controlled by Gen5 software (Gen5 version 2.04 BioTek Inst. Inc, Winooski, VT, USA). Then, 100 µL of sample were added to 745 µL of a methanolic solution of daily-working DPPH^•^ solution at room temperature for 1 h in the dark. Then, 300 µL were taken out and placed in 96-well microplates to measure their total absorbance. IC_50_ values were calculated as the concentrations (µmol/L) that inhibited 50% of the DPPH^•^ radicals in the reaction, where radical scavenging activity was calculated as follows:

$$DPPH\;radical\;scavenging\;\left(\%\right)=\frac{Abs_{control}-Abs_{sample}}{Abs_{control}}*100$$

where *Abs_control_* is the absorbance of DPPH^•^ radical in methanol and *Abs_sample_* is the absorbance of an DPPH^•^ radical solution mixed with sample. All determinations were performed in triplicate (*n* = 3).

Regarding ABTS^•+^ assay, the antioxidant activity was investigated by their ability to scavenge the ABTS^•+^ cation radical using the method proposed by Re et al. [69]. Briefly, ABTS^•+^ was produced by reacting 2.5 mL of 7 mM ABTS^•+^ stock solution with 44 µL of 2.5 mM potassium persulfate, allowing the mixture to stand in the dark at room temperature for 16 h before use. The ABTS^•+^ solution (1 mL) was diluted with 70 mL of 5 mM phosphate buffered saline (PBS, pH 7.4) to an absorbance of 0.70 ± 0.02 at 734 nm. The reaction mixture was placed in 96-well microplates containing 10 µL sample/standard and 300 µL of reagent; then, the reaction was incubated in the dark at room temperature for 45 min. The absorbance was measured at 734 nm using a microplate reader (Synergy HT, BioTek Instruments, Winooski, VT, USA) controlled by Gen5 software (Gen5 version 2.04 BioTek Inst. Inc). Appropriate solvent blanks were run in each assay. IC_50_ values were calculated as the concentrations (µmol/L) that inhibited 50% of the ABTS^•+^ in the reaction, where radical scavenging activity was calculated as follows:

$$ABTS\;radical\;scavenging\;\left(\%\right)=\frac{Abs_{control}-Abs_{sample}}{Abs_{control}}*100$$

where *Abs_control_* is the absorbance of ABTS^•+^ radical in PBS solution and *Abs_sample_* is the absorbance of an ABTS^•+^ radical solution mixed with sample. All determinations were performed in triplicate (*n* = 3).

### 4.6. Statistical Analysis

All determinations were carried out at least three times. One-way ANOVA and Tukey’s multiple range test at a 5% level of significance were also evaluated. To find IC_50_ values (the concentration of agonist that gives a response halfway between Bottom and Top), non-linear regression analysis fitting ([Inhibitor] vs. response–variable slope and four parameters) was done using GraphPad Prism V. 6.0 (GraphPad Soft. Inc., La Jolla, CA, USA). The IC_50_ model was:

$$Y=Bottom+\frac{Top-Bottom}{1+\frac{X^{HillSlope}}{IC50^{HillSlope}}}$$

where *X* and *Y* are the data table. HillSlope describes the steepness of the family of curves. A HillSlope equal to −1.0 was chosen (standard steepness). Top and Bottom are plateaus in the units of the *Y* axis.

## 5. Conclusions 

An effective method for the large-scale separation of procyanidins was developed using preparative HPLC with C18 and diol stationary phase columns. In addition, comparison of preparative HPLC vs. classical fractionation of procyanidins was also studied. Results confirm that preparative HPLC has many advantages compared to solid phase separation and Sephadex LH-20 gel permeation. All methodologies studied allowed good separation of alkaloids and catechins. In addition, enriched oligomeric samples could be obtained using classical approaches and preparative reversed phase HPLC. Better separation of individual PCs according to the degree of polymerization was only obtained using preparative-HPLC with a diol column. This procedure was demonstrated to be, by far, more efficient and robust than the use of C18 stationary phase. For instance, using a diol column, the fractionation time was not only reduced to 18 min but also the solvent consumption decreased 71–93% compared to the other fractionating methods. In this sense, this procedure could be useful for the recovery of procyanidins from similar matrices such as tea, grape, berries, and apple but further studies should be performed. In addition, this work showed that high antioxidant effectiveness against radicals is enhanced by long-chain procyanidins followed by oligomers and monomers.

## Figures and Tables

**Table 1:** Yield, purity, characterization, and structural mass pattern for each procyanidin fraction from cocoa polyphenolic extract using obtained by preparative reversed phase.

F	Yield (%)	Type	Number Isomers	Minor Compounds	Major Compound					
					Molecule / RT (min)	Contribution (%)	CE (eV)	Mode	MS	MS/MS
0	0.97	Alkaloid	-	-	Theobromine (6.800)	>95	20	[M + H]^+^	181.0742	138.0663, 110.0713, 67.0291
I	0.44	Dimers	3	Traces of EC	Dimer B2 (10.850)	>95	20	[M − H]^−^	577.1342	407.0782, 289.0723, 125.0243
II	0.92	Catechin	-	Traces of Dimer	(–)-Epicatechin (11.890)	>95	20	[M − H]^−^	289.0717	245.0824, 151.0400, 109.0302
III	0.35	Alkaloid	-	Traces of EC	Caffeine (15.210)	>95	20	[M + H]^+^	195.0882	138.0668, 110.0712, 69.0449
IV	0.58	Trimers	4	Traces of dimer, tetramer and pentamer	Trimer C1 (20.430)	≤80	20	[M − H]^−^	865.1960	577.1363, 425.0903, 287.0569
V	0.39	Mix PCs	-	Traces of dimers. Trimers, and pentamers	Tetramer (22.256)	≤54	20	[M − H]^−^	1153.2583	863.1841, 575.1186, 287.0558
VI	0.21	Mix PCs	-	Tetramers, and heptamers	Hexamer (27.430)	≤60	20	[M − 2H]^2−^	864.1864	779.6594, 577.1344, 289.0738
VII	0.07	Mix PCs	-	Mixture of PCs with DP 3-7	Heptamer (32.765)	≤15	20	[M − 2H]^2^	1008.2167	863.1792; 575.1202; 289.0724
VIII	0.06	Mix PCs	-	Mixture of PCs with DP 3-9	Octamer (36.720)	≤12	20	[M − 3H]^3^	768.1671	575.1192; 413.0882; 289.0723
IX	0.75	Mix PCs	-	Mixture of PCs with DP 3-9	Trimer to Pentamer (42.800)	NA	-	-	-	-

Yield is expressed as mg fraction/mg cocoa extract. MS and MS/MS fragments for main compounds. F—fraction number; RT—retention time; CE—collision energy; Mix PCs—mixture of procyanidins.

**Table 2:** Yield, characterization, and structural mass pattern for each procyanidin fraction from cocoa polyphenolic extract using preparative diol column.

F	Yield (%)	Type	Number Isomers	Minor Compounds	Major Compound					
					Molecule / RT (min)	Contribution (%)	CE (eV)	Mode	MS	MS/MS
0	1.26	Alkaloids	-	Traces of catechins	Theobromine	71.0	20	[M + H]^+^	181.0724	138.0660, 110.0715, 83.0610
					Caffeine	29.0	20	[M + H]^+^	195.0881	138.0663, 110.0711, 69.0450
I	1.41	Catechins	-	Traces of dimer	(+)-Catechin	7.9	20	[M − H]^−^	289.0727	245.0820, 203.0713, 125.0238
					(–)-Epicatechin	91.2		[M − H]^−^	289.0742	245.0830, 203.0720, 125.0246
II	0.64	Dimers	8	Traces of EC, and trimer	Dimer B2 (5.194)	62.3	20	[M − H]^−^	577.1358	425.0879, 289.0715, 125.0238
III	0.89	Trimers	16	Traces of EC, and dimer	Trimer C1 (7.000)	59.3	20	[M − H]^−^	865.1993	575.1205, 287.0566, 125.0241
IV	0.79	Tetramers	14	Traces of EC	Tetramer (7.678)	52.2	40	[M − H]^−^	1153.2624	865.1971, 575.1197, 287.0564
V	0.63	Pentamers	17	Traces of EC and dimer	Pentamer (8.376)	63.7	40	[M − H]^−^	1441.3170	1153.2597, 863.1834, 575.1196
VI	0.60	Hexamers	18	Traces of EC	Hexamer (9.138)	51.3	40	[M − H]^−^	1729.8774	1153.2555, 863.1834, 575.1188
VII	0.56	Heptamers	19	Traces of EC	Heptamer (9.611)	69.9	20	[M − 2H]^2−^	1008.7263	863.1849, 577.1347, 287.0568
VIII	0.16	Octamers	17	Traces of EC	Octamer (10.087)	68.9	40	[M − 2H]^2−^	1152.7555	865.2007, 575.1195, 287.0562
IX	0.29	Nonamers	15	Traces of EC	Nonamer (10.476)	60.0	40	[M − 2H]^2−^	1296.7871	1152.2519, 863.1829, 287.0569
X	0.23	Decamers	NA	Traces of EC	Decamer (12.598)	NA	40	[M − 2H]^2−^	1440.8102	1151.2432, 863.1846, 287.0578

Yield is expressed as mg fraction/mg cocoa extract. F—fraction; NA—not available; F—fraction number; RT—retention time; CE—collision energy. Exact elucidation of molecules from Fractions 0–III were confirmed with commercial standards.
